# Administration of Salicylic Acid

**Published:** 1878-02

**Authors:** 


ADMINISTRATION OF SALICYLIC ACID.—Dr. GUENEAU DE MUSSY recommends the administration of salicylic acid by dissolving it in a syrup of gum by the aid of ten times its weight of brandy, and adding to it a little lemon juice.

